# Changes in the Oxidative Stress Status of Dogs Affected by Acute Enteropathies

**DOI:** 10.3390/vetsci9060276

**Published:** 2022-06-06

**Authors:** Alessia Candellone, Flavia Girolami, Paola Badino, Watanya Jarriyawattanachaikul, Rosangela Odore

**Affiliations:** Department of Veterinary Sciences, University of Torino, 10095 Grugliasco, TO, Italy; alessia.candellone@unito.it (A.C.); flavia.girolami@unito.it (F.G.); watanya.jarriyawattanachaikul@unito.it (W.J.); rosangela.odore@unito.it (R.O.)

**Keywords:** acute diarrhea, oxidative stress, antioxidant status, dog

## Abstract

Canine acute enteropathies (AE) are common morbidities primarily managed with supportive therapy. However, in some cases, unnecessary courses of antibiotics are empirically prescribed. Recent studies in humans have hypothesized the use of antioxidants as a possible alternative and/or support to antimicrobial drugs in uncomplicated cases. Considering the global need to reduce the antibiotic use, the aim of the study was to compare the oxidative burden of the diarrhetic population to that of healthy dogs. Forty-five patients suffering from uncomplicated acute diarrhea (AD) and 30 controls were screened for clinical and biochemical parameters, and serum redox indices (reactive oxygen metabolites, dROMs; serum antioxidant capacity, SAC; oxidative stress index, OSi). The average levels of dROMs in AD dogs were significantly higher (*p* < 0.001) than in healthy dogs, while SAC did not significantly differ between the two groups. However, the OSi values (ratio between dROMs and SAC) significantly increased (*p* < 0.001) in AD dogs compared to controls. The study demonstrates that canine AD could induce redox imbalance. Although its role in the etiopathogenesis and evolution of the disease should be further investigated, our results suggest that the improvement of the patient oxidative status, possibly through the dietary administration of antioxidants, could support the management of canine AE, reducing the use of antibiotics.

## 1. Introduction

Canine acute enteropathies (AE) are common disorders with a complex etiology including infectious diseases, stressful events, dietary indiscretion, metabolic disturbances, and other gastrointestinal tract inflammatory stimuli [1]. The predominant clinical manifestation of AE is acute diarrhea (AD), which is defined as the abrupt onset of 3 or more loose feces per day lasting no longer than 7 days [2]. Although most AD episodes are self-limiting and symptomatic treatment should represent the recommended management of the uncomplicated disease, AE are still a frequent reason for the empirical prescription of antibiotics [3,4]. Very little information is available about the clinical benefits of antimicrobial treatment in uncomplicated AD, and their administration should be limited to dogs with signs of sepsis [3]. Limitation to the use of antimicrobials is justified by the possible disruption of the intestinal microbiota and the selection of antibiotic-resistant bacterial strains in both dogs and cohabitant species, including humans [5,6,7]. As a matter of fact, the increasing need to reduce the overuse of antibiotics in veterinary clinics to avoid the potential zoonotic transmission of resistant organisms supports the search for alternative therapeutical approaches [4].

In humans and in laboratory animal models acute and chronic gastrointestinal diseases are characterized by an altered redox homeostasis due to either an over production of reactive oxygen species (ROS) or a deficiency of counteracting antioxidant systems [8,9,10,11]. However, clinical trials evaluating the antioxidant status and the role played by oxidative stress in dogs affected by AD are scattered and mainly focused on AE of viral etiology [12,13]. Panda et al. [12] investigated oxidative stress indices in dogs suffering from parvoviral gastroenteritis and concluded that the activities of the antioxidant enzymes (i.e., catalase and superoxide dismutase) were altered compared to healthy subjects. Moreover, such alterations were more pronounced in canine parvovirus-positive cases compared to diarrhetic parvovirus-negative patients. Accordingly, Gaykwad et al. [13] found evidence for the supportive use of the antioxidant compound *N*-acetylcysteine in the treatment of dogs diagnosed with parvoviral infection. The *N*-acetylcysteine treatment significantly improved the white blood cells (WBC) count and the glutathione S-transferase activity, while reducing the nitrates and malondialdehyde levels, in parvovirus-infected dogs compared to non-supplemented patients.

Even though some evidence of an altered redox homeostasis during both acute and chronic enteropathies has been reported both in humans and in several animal models, no information about the OS status of dogs suffering from AE others than those of viral etiology is available. Moreover, the use of antioxidants in uncomplicated enteropathic patients has been suggested as a possible alternative to drugs [4]. For these reasons, we performed a clinical trial enrolling dogs affected by nonspecific AD in order to: (i) evaluate the redox status of the animals; (ii) compare the oxidative burden of the diarrhetic population to that of healthy dogs. The results of the study could set the stage for introducing antioxidants supplementation in the diet of dogs affected by AE.

## 2. Materials and Methods

### 2.1. Animals

Dogs with AD presented to the “Struttura Didattica Speciale Veterinaria (SDSV)” of the University of Turin and other referral clinics throughout Italy were enrolled for participation in an observational cross-sectional clinical trial from June 2019 to June 2021. The study was approved by the local ethical committee (n. 248) and a written informed con-sent was obtained from all owners.

Inclusion criteria were defined according to Langlois et al. [14]. Briefly, dogs ageing more than 6 months, with a body weight between 4 and 40 kg, regularly vaccinated and dewormed, and presenting with an AD of less than 7 days in duration, were enrolled. Due to the possible interference with the patient redox status, the fulfilment of one of the following conditions was considered as an exclusion parameter: (i) chronic gastrointestinal diseases characterized by active symptoms; (ii) non-gastrointestinal diseases causing gas-trointestinal signs (e.g., hypocortisolism) and/or concurrent systemic diseases (e.g., chronic liver disease, chronic kidney disease IRIS Stage 1–4, congestive heart failure, systemic neoplasia, immune-mediated disease); (iii) suspected nutritional deficiencies; (iv) treatment with anti-inflammatory and/or antimicrobial drugs during the last 15 days; and (v) antioxidant supplementation or commercial diets enriched with patented antioxidant formula [15,16].

A representative number of healthy dogs were recruited in the same period as a control group. The dogs were classified as diarrhetic or healthy according to their history, physical examination, results of complete blood biochemical profile, and fecal analysis, including Giardia spp. and Parvovirus test. Serum cortisol concentration, abdominal radio-graphic findings and/or abdominal ultrasound were also performed in diarrhetic dogs when deemed necessary for diagnosis. Dogs with acute hemorrhagic or non-hemorrhagic diarrhea, with or without mucus, that did not have evidence of gastro-intestinal parasitism, Giardia spp. infection, or parvoviral enteritis, were considered to suffer acute nonspecific diarrhea and were included in Group AD. Dogs resulting as clinically and biochemically healthy, without signs of gastrointestinal parasites, were recruited as control group (Group C).

Body condition score (BCS) was measured according to the 1–9 WSAVA point-scale [17], while fecal score was assessed according to the 1–7 Purina Fecal Scoring system [18]. Severity of illness of acute diarrhetic patients was determined by a modified-Vesikari Scoring System [19] (mVSS) (Table 1). Briefly, four parameters (i.e., diarrhea, vomiting, severity of dehydration and treatment) were scored according to their severity from 1 (less severe) to 3 (more severe). Partial scores obtained from each parameter were then summed together to obtain the severity rating scale. Patients with a severity rating scale less than 5 were considered as mildly affected; patients with a severity rating scale from 6 to 10 were classified as moderately affected; and patients with a total score more than 11 were judged as severely affected.

### 2.2. Hematobiochemical Analyses

All dogs were fasted 10–12 h prior to blood sampling. Hematology tubes containing EDTA were immediately analyzed to obtain WBC, red blood cells (RBC) and hematocrit (Hct) values. Tubes without anticlotting agents were centrifuged for serum collection. Serum was divided into two aliquots: the first aliquot was used for immediate determination of biochemical analysis (albumin, ALB; total protein, PT; blood urea nitrogen, BUN; creatinine, CREA; alanine-amino transferase, ALT; glucose and electrolytes, such as sodium, potassium and chlorum); the second aliquot was stored at −80 °C until analysis of the redox status [20].

### 2.3. Redox Status Assessment

Determinable reactive oxygen metabolites (dROMs) were quantified using the dROMs Test (Diacron International Srl, Grosseto, Italy) as an indicator of oxidative stress due to free radicals. Briefly, in a biological sample reactive oxygen metabolites (primarily hydroperoxides) are able to generate alkoxyl and peroxyl radicals, in the presence of iron released from plasma proteins by an acidic buffer, according to the Fenton reaction. Such radicals can then oxidize an alkyl-substituted aromatic amine (*N*,*N*-dietylparaphenylendiamine), thus producing a pink-colored derivative that is photometrically quantified at 505 nm [21]. The d-ROMs concentration is directly proportional to the colour intensity and is expressed as Carratelli Units (1 CarrU = 0.08 mg hydrogen peroxide/dL).

The Serum Antioxidant Capacity (SAC) was measured by the OXY-Adsorbent test (Diacron). The assay evaluates the ability of the serum barrier to neutralize the oxidative action induced by an hypochlorous acid solution (HClO). The method was modified from Jansen and Ruskovska [22]. Briefly, 2 µL of serum samples diluted 1:100 (*v*/*v*) with MilliQ-water were incubated with 200 µL of HClO solution for 10 min at 37 °C in a 96-well plate. Then, 2 µL of chromogenic reagent (*N*,*N*-diethyl-*p*-phenylenediamine) was added. The intensity of the colored complex is inversely related to the total antioxidant capacity in serum. The absorbance values were measured at 505 nm using the Glomax Multi detection system spectrophotometer. Data were expressed as µmol of HClO/mL.

The Oxidative Stress index (OSi) was defined as the ratio of the dROMs to SAC values. Specifically, OSi (arbitrary units, A.U.) = dROMs (CarrU)/SAC (mmol of HClO/mL) [15,23].

### 2.4. Statistical Analysis

To neutralize the possible interference of breed size, age, gender, and BCS in comparing dROMs, SAC and OSi values between Group AD and C, different subgroups were preliminarily identified. The following criteria were adopted: breed size subgroups were classified as mini and small breed dogs (i.e., Chihuahua, miniature Pincher, French bulldog, Poodle or adult dogs weighting < 10 kg), medium breed dogs (i.e., English setter, Breton, or adult dogs weighting from 11 kg to 25 kg), and large and giant breed dogs (i.e., Labrador and Golden retrievers, Great Dane, or adult dogs weighting > than 25 kg). Age-subgroups were classified as young (dogs within the age interval of 6 months to adulthood age, according to breed size), adult (dogs from adulthood to maturity, according to breed size) and mature (dogs in the last third of their lifespan, according to breed size). Sex-subgroups included female, neutered female, male and castrated male dogs. BCS-subgroups were classified according to WSAVA guidelines [17] as ideal (dogs with a BCS of 5 out of 9), under ideal (dogs with a BCS of 1–3 out of 9), and over ideal (dogs with a BCS of 6–9 out of 9). To identify correlations between oxidative stress parameters and the considered covariates (breed size, age, sex, BCS, and selected haemato-biochemical parameters [RBC, WBC, Hct, BUN, Crea, PT, Alb, ALT, GLU, Na, K, Cl]), a preliminary statistical analysis was performed. A quantile multivariate regression model was applied using the software StataCorp. 2015 (Stata: Release 14. Statistical Software. College Station, TX, USA: StataCorp LP). The statistical significance was set at 5% level (*p* < 0.05). Age, sex, breed, size and BCS did not significantly influenced dROMs, SAC and OSi parameters, when comparing subgroups of Group AD and Group C (*p* > 0.05 for all considered covariates; data not shown, although available upon request). No significant correlation was also appreciated between haemato-biochemical parameters and redox indices (*p* > 0.05 for all investigated parameters; data not shown, although available upon request). Given the above, a possible interference between d-ROMs, SAC, OSi and the identified covariates was considered irrelevant. Thus, d-ROMs, SAC and OSi results were compared between Group AD and C (without subgroups). Moreover, patients belonging to Group AD were further subgrouped on the basis of mVSS into mild, moderate, and severe cases according to Table 1.

To select the appropriate parametric or non-parametric tests for the statistical analysis, haemato-biochemical parameters and redox status markers were checked for normal distribution by Shapiro–Wilk test. Haemato-biochemical parameters resulted as normally distributed, thus an unpaired t-test with the Welch’s correction was applied to compare group AD and C. Redox indices were not normally distributed, thus Mann–Whitney and Kruskal–Wallis tests (followed by Dunn’s multiple comparisons) were used to compare Group AD and C, and subgroups (mild, moderate, and severe cases) of group AD, respectively. Differences were considered statistically significant when the two-sided *p* value was <0.05. Statistical analysis was performed using GraphPad Prism 9.0 (GraphPad software, CA, USA). In order to visualize the data, box-whisker plots were utilized.

## 3. Results

Thirty (30) healthy dogs and 60 ill dogs were eligible for the enrolment, but 13 sick cases were excluded from the study because of gastrointestinal parasitism (*n* = 3), concurrent disease (*n* = 2, foreign body ingestion and acute pancreatitis), or inability to obtain sufficient feces or serum for analysis (*n* = 8). Furthermore, two serum samples of the ill group stored for redox status evaluation were discarded before analysis because of hemolysis. A total of 75 dogs met all inclusion criteria, including 45 patients suffering from AD (Group AD) and 30 controls (Group C). Demographics and baseline characteristics of Group AD and C are summarized in Table 2. Diseased patients were mainly represented by intact males equally distributed between breed sizes, with a mean age of 2.3 years, a mean body weight of 23.5 kg, and a BCS of 4/9. No differences were recorded about the demographic characteristics between AD and C groups. By contrast, the fecal score value was higher in group AD compared to controls (6 vs. 2, *p* < 0.032), in line with the inclusion criteria. With few exceptions (Na^+^, Cl^−^ and K^+^), the haemato-biochemical parameters were in the normal range for both groups. However, group AD had significantly higher hematocrit, and RBC and WBC count (*p* < 0.01, *p* < 0.01 and *p* < 0.0001, respectively) than healthy dogs, and increased serum levels of PT, BUN and ALT (*p* < 0.05, *p* < 0.01 and *p* < 0.0001, respectively). As regards the mVSS, Group AD had an average score of 9.8, indicating a moderate severity index.

**Figure 1:**
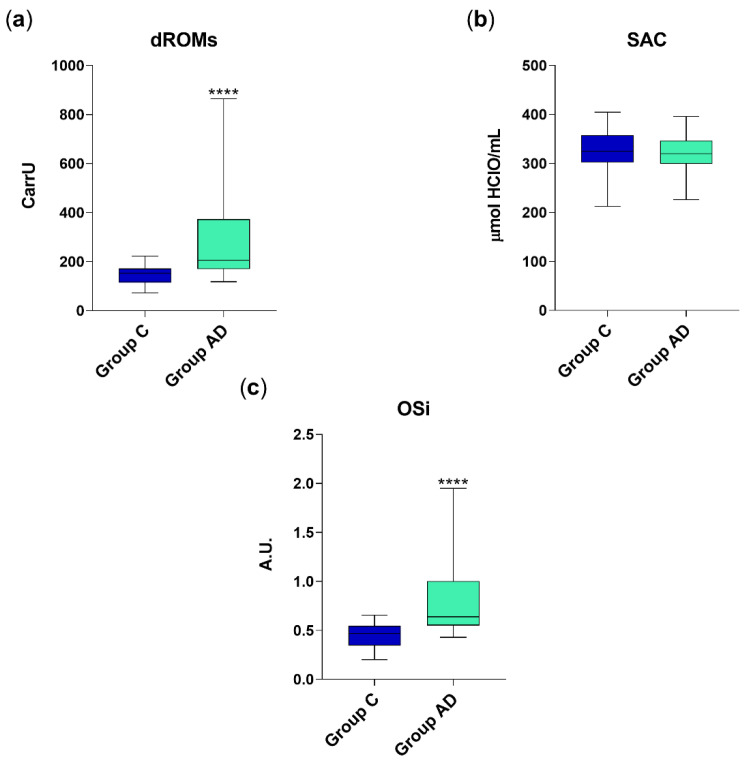
dROM (**a**), SAC (**b**) and OSi values (**c**) in group C (healthy animals, *n* = 30) and AD (diarrhetic dogs, *n* = 45). Data are represented as boxplot showing median and interquartile range and analyzed by Mann–Whitney test. Statistical differences with respect to group C are reported (**** *p* < 0.0001). A.U. = arbitrary units.

The average levels (mean ± standard deviation) of serum dROMs in AD dogs (290 ± 191.5 CarrU) were significantly higher (*p* < 0.001) than those of healthy animals (146.46 ± 42.97 CarrU) of approximately 2-fold (Figure 1a). However, the SAC did not significantly differ between the two groups (322.9 ± 37.4 μmol HClO/mL in Group AD vs. 325.5 ± 37.5 μmol HClO/mL in Group C), as depicted in Figure 1b. Despite that, being the oxidative stress burden increases in AD patients, the OSi values resulted significantly increased (*p* < 0.001) in group AD (0.88 ± 0.51) as compared to control group (0.44 ± 0.13) (Figure 1c).

**Figure 2:**
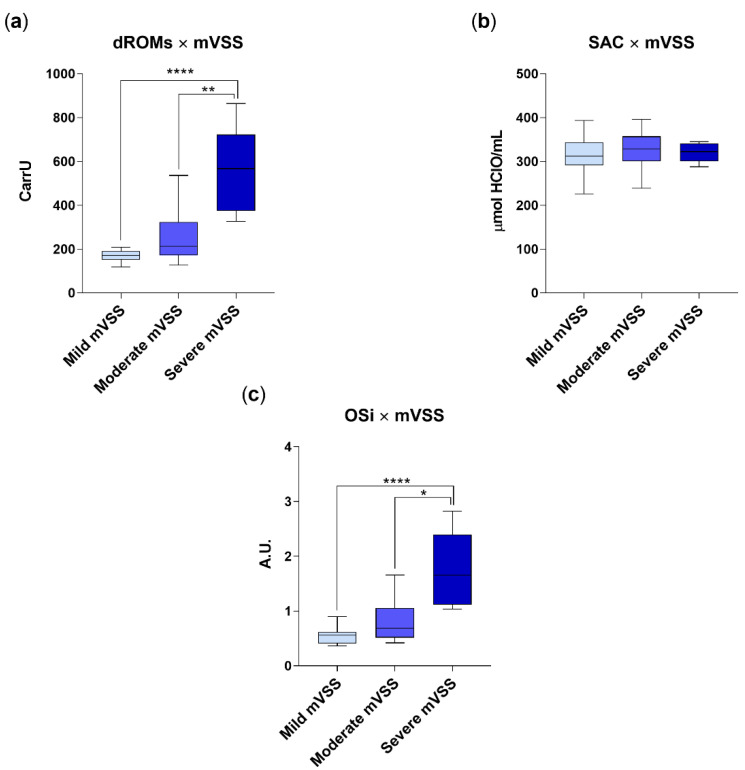
dROM (**a**), SAC (**b**) and OSi values (**c**) in group AD (diarrhetic dogs, *n* = 45), categorized on the basis of mVSS. Mild mVVS subgroup includes dogs with a mVVS score < 5; moderate mVVS subgroup includes dogs with a mVVS score from 6 to 10, and severe mVVS subgroup includes dogs with a mVSS score > 11. Data are represented as boxplot showing median and interquartile range and analyzed by Kruskal–Wallis test followed by Dunn’s multiple comparisons. Statistical differences between groups are reported (* *p* < 0.05; ** *p* < 0.001; **** *p* < 0.0001).

Correlation between mVSS subgroups, oxidative stress and redox status markers is depicted in Figure 2. Briefly, dogs included in the severe mVVS subgroup had a significantly higher redox burden (dROMS: 562.3 ± 184.9 CarrU; OSi: 1.7 ± 0.66; *p* < 0.001) as com-pared to patients that were moderately (dROMS: 241.6 ± 108 CarrU; OSi: 0.8 ± 0.36) or mildly (dROMS: 169.1 ± 26.1 CarrU; OSi: 0.54 ± 0.14) affected. By contrast, no differences emerged when assessing SAC among the three subgroups of severity (mild mVSS subgroup: SAC value of 314.9 ± 42.4 μmol HClO/mL; moderate mVSS subgroup: SAC value of 328.4 ± 39.5 μmol HClO/mL and severe mVSS subgroup: SAC value of 320.1 ± 21.2, μmol HClO/mL, respectively).

None of the other considered variables (e.g., sex, age, BCS, biochemical parameters, etc.), as previously mentioned, were significantly correlated with the measured oxidative parameters.

## 4. Discussion

The present study represents the first attempt to investigate the oxidative status in dogs suffering an acute nonspecific enteropathy. While the contribution of oxidative stress in chronic gastrointestinal disorders is well established [9,24,25], few data are available on the acute disease. Elevated levels of ROMs have been detected in humans affected by inflammatory bowel disease (IBD) and ulcerative colitis (UC), as well as in murine models with acute and chronic colitis [26]. It is therefore reasonable to argue that high levels of ROMs may also play a key role in the pathogenesis of acute enteropathies in dogs, potentially leading to mucosal damage and/or delay in recovery time.

The cohort of diarrhetic patients enrolled in the present trial was in line with the population described by Langlois et al. [14], which inspired the definition of our inclusion criteria. However, the direct comparison of the illness severity among the two canine populations is not applicable, as the mVSS was here used for the first time. This clinical score was originally adopted for predicting the viral or bacterial pathogens in pediatric gastroenteritis [27]. Children were divided into bacterial, viral and nonspecific groups, according to the pathogens detected using a PCR test. Patients in the bacterial group had significantly higher Vesikari-scoring system as compared to those in the viral and in the nonspecific group [27]. In our study, only canine patients with AD that did not have evidence of gastrointestinal parasitism, Giardia spp. infection, or parvoviral enteritis were included in the AD Group, resembling the “nonspecific group” of the human study. However, in our cohort the presence of a non-diagnosed specific bacterial infection could not be completely ruled out, as a routinary fecal culture and/or a PCR assay for specific bacteria (e.g., *Campylobacter* spp., *Salmonella* spp.) was not performed. Hence, translational conclusions between pediatric and canine population should be made carefully.

As regards the hematobiochemical parameters, dogs enrolled in the Group AD showed a profile consistent with the moderate severity of the disease [1]. In details, increased Hct (57.8%) and serum levels of PT were likely due to a mild dehydration, as already reported [28,29]. Indeed, a recent study including 108 dogs with acute hemorrhagic diarrhea syndrome (AHDS) showed that the median Hct at presentation was 57%, with a range between 33% and 76%. The leukocytosis with neutrophilia and the monocytosis highlighted in our trail could be attributable to the stress response (“stress leukogram”), as described for dogs affected by AHDS [30]. Moreover, in these subjects the extensive mucosal damage can determine a mild left shift that is also observed in about 50% of dogs with an uncomplicated clinical course and without signs of sepsis [30]. However, such hematological parameter was not included in our evaluation, then a comparison with the previous literature could not be made. Despite a severe dehydration, most dogs with AHDS do not usually experience prerenal azotemia [30]. Accordingly, BUN serum levels in Group AD were within the reference interval, even if higher as compared to control group. Finally, we detected hyponatremia, hypokalemia, and hypochloremia in ill dogs, that can be interpreted as the typical electrolyte imbalance in course of acute enteropathy, as previously described by other authors [31,32,33].

The oxidative stress values observed in the control group of the present study were slightly higher than those measured by Pasquini et al. [20]. The authors reported that the dROMs values in healthy Labrador dogs ranged from 56 to 91 CarrU. Our healthy population, composed of a variety of small, medium and large breed dogs had a mean dROMs value of 146.46 ± 42.97 CarrU. Species and breed-related differences cannot be excluded. Indeed, in our study the small breed subgroup size might have represented a bias when trying to apply a quantile multivariate regression model to identify breed-related differences.

In the gastrointestinal tract, infections and inflammatory processes act as oxidative stressors by stimulating the production of cytokines which, in turn, increase ROS production [34]. Besides causing lipid peroxidation and cell damage, excessive ROS are associated with intestinal dysbiosis [24]. In our study, oxidative stress indices and redox burden (dROMs and OSi, respectively) were increased in the diarrhetic population compared to healthy subjects. Evidence of increased ROM production and/or redox imbalance in some acute and chronic gastrointestinal diseases has been documented in different animal species [10,11,12,13,35,36]. As already mentioned, few studies have been performed in the canine patient. Interestingly, Panda et al. [12] demonstrated an increase in oxidative stress indices in dogs suffering from gastroenteritis due to parvoviral infection. Indeed, the Authors found modification of peripheral markers both in moderately and severe affected animals. Our results seem to confirm that redox imbalance could play a role in the etiopathogenesis and evolution of acute non-viral uncomplicated canine AD. Moreover, in the present study dROMs and OSi parameters were correlated with the severity of the clinical presentation, being significantly higher in dogs with a mVSS score > than 6.

On the other hand, the potential role of oxygen reactive species in the pathogenesis of AE should be considered in the light of the endogenous antioxidant mechanisms limiting ROS-induced tissue damage [34]. As concerns the SAC evaluation, in our study no significant difference was observed between healthy and affected dogs, suggesting a still effective residual capacity to counteract the oxidative stress stimuli. Nevertheless, it has been already demonstrated that oxidative stress and redox unbalance may contribute to damaging the gut barrier in the course of AD [4], and the hypothesis that a persistent redox imbalance can trigger chronic gastrointestinal disease later in life has been recently investigated [37]. The hypothesis is supported by the observation that oxidative stress occur-ring during acute inflammation can lead to dysbiosis by reducing the microbial diversity in the gut and by promoting the overgrowth of specific microbial taxa [38]. In experimental animal models it has been demonstrated that soon after the onset of inflammation a redox imbalance could occur, leading to the depletion of near the 80% of gut microbiota [10,26].

In line with what observed by Panda et al. [12], there was no effect of sex and age on the oxidative stress markers in our canine patients. By contrast, some findings in humans and rats seem to suggest gender differences with a greater oxidative stress in males compared with females [39,40]. A possible mechanism underlying the observed sex specific differences relates to the beneficial effects exerted by 17β-estradiol. The hormone acts as a transcription factor by inducing the expression of different antioxidant proteins [41]. On the other hand, the mitochondrial estrogen receptors can mediate reduced ROS production in response to estrogen. From this point of view, present data are not yet adequate to allow definitive conclusions in canine patients and further studies on a wider cohort of dogs need to be performed to highlight the possible role played by age and gender on the individual capability to cope with redox unbalance and illness status.

Our findings need to be considered in the light of some limitations. The first one is the non-standardized diet. Even though patients receiving commercial diets enriched with patented antioxidant formula were not involved in the trial, we cannot exclude that vegetal ingredients naturally containing polyphenols or free radical scavengers could have interfered with the redox status. Moreover, a possible bias could be the unknown etiology of AD. Finally, ill dogs were enrolled in the study from the moment they were referred to the clinic, and not exactly from the first diarrhetic episode. In this time-window, undetected variations of the oxidative stress indices could have occurred; such an event may be responsible for the high interindividual variability of the d-ROMs values in Group AD.

## 5. Conclusions

The present study indicates for the first time that canine patients suffering from AD could experience redox imbalance. Despite the exact mechanisms underlying such event are still to be elucidated, our results open the way to new strategies for the treatment of GI disorders, based on antioxidants as potential adjuvants. Indeed, the administration of natural antioxidant molecules may represent a valuable therapeutic and prophylactic tool to counteract the short and long-term damage of the gut barrier occurring in acute gastrointestinal disturbances, possibly reducing the prescription of antimicrobial drugs in uncomplicated cases. Clinical trials supporting the efficacy of antioxidant therapy during canine acute enteropathies are therefore advisable.

## Figures and Tables

**Table 1:** Modified-Vesikari Scoring System (mVSS).

Parameters	1	2	3
**Diarrhoea**			
Maximum number stool/day	1–3	4–5	More than 6
Characteristics	No mucous nor blood	Mucous	Blood
Fecal score	4	5–6	7
**Vomiting**			
Maximum number per day	1–2	3–4	More than 5
**Severity of dehydration (%)**	N/A	1–5	More than 6
Treatment	Symptomatic. No parenteral rehydration needed. No hospitalization	Symptomatic + rehydration therapy. Day-hospital treatment for parenteral rehydration	Symptomatic + rehydration therapy + supportive (i.e., assisted feeding). Hospitalization needed
Severity rating scale *	**<5 (Mild)**	**6–10 (Moderate)**	**More than 11 (Severe)**

* modified from [19].

**Table 2:** Demographics and baseline characteristics of Group AD and C.

Parameters	Group AD	Group C	
**Signalment and clinical scores**			
Age (years)	2.3 ± 5.3	2.5 ± 8	
Sex	52% Male 48% Female	49% Male 51% Female	
Breed size	42% Small and mini 20% Medium 38% Large and Giant	35% Small and mini 35% Medium 30% Large and Giant	
Predominant breeds	French Bulldog (12%) and Labrador Retriever (14%)	Mixed breed dog (30%) and Golden Retriever (15%)	
Body weight (kg)	23.5 ± 12.1	26 ± 10	
BCS	4/9	5/9	
Fecal score	6	2	
**mVSS**	9.8	N/A	
Mild mVSS (number of dogs)	15	N/A	
Moderate mVSS (number of dogs)	21	N/A	
Severe mVAA (number of dogs)	9	N/A	
**Haemato-biochemical parameters**			Normal range
Alb (g/dL)	3.3 ± 0.3	3.2 ± 1.1	3.0–3.7
PT (g/dL)	6.3 ± 1.2 *	5.9 ± 0.9	5.7–7.1
BUN (mg/dL)	31 ± 15 **	22 ± 10	19–45
CREA (mg/dL)	0.9 ± 0.5	1 ± 0.3	0.76–1.24
Glucose (mg/dL)	117 ± 11	101 ± 23	83–125
ALT (IU/L)	91 ± 10 ****	78 ± 12	17–108
Na^+^ (mEq/L)	141 ± 8 *	150 ± 3	143–151
K^+^ (mEq/L)	3.8 ± 0.5 *	4.2 ± 0.5	3.9–4.9
Cl^−^ (mEq/L)	104 ± 4 *	111 ± 2	109–117
RBC (10^6^/μL)	7.67 ± 1.5 **	6.5 ± 1.4	6.13–8.52
Hct (%)	57.9 ± 5 **	37 ± 6	42–58
WBC (10^3^/μL)	10.78 ± 3.6 ****	5.78 ± 4.9	4.7–11.15

Alb: albumin; ALT: alaninoaminotransferase; BUN: blood urea nitrogen; CREA: creatinin; Hct: haematocrit; mVSS: modified Vesikari Scoring system; PT: total protein; RBC: red blood cells; WBC: white blood cells. For continuous variables, data are expressed as mean ± standard deviation; for non-continuous parameters only mean is provided. Continuous data were analyzed by unpaired t-test with the Welch’s correction. * *p* < 0.05; ** *p* < 0.01; **** *p* < 0.0001.

## Data Availability

The raw data supporting the conclusions of this article will be made available by the authors, without undue reservation.
